# The Role of Chondrocyte Morphology and Volume in Controlling Phenotype—Implications for Osteoarthritis, Cartilage Repair, and Cartilage Engineering

**DOI:** 10.1007/s11926-019-0837-6

**Published:** 2019-06-15

**Authors:** Andrew C. Hall

**Affiliations:** Deanery of Biomedical Sciences, University of Edinburgh, Hugh Robson Building, George Square, Edinburgh, Scotland EH8 9XD UK

**Keywords:** Chondrocyte, Volume regulation, Morphology, Hypertrophy, Fibroblast, Phenotype

## Abstract

**Purpose of Review:**

Articular chondrocytes are exclusively responsible for the turnover of the extracellular matrix (ECM) of hyaline cartilage. However, chondrocytes are phenotypically unstable and, if they de-differentiate into hypertrophic or fibroblastic forms, will produce a defective and weak matrix. Chondrocyte volume and morphology exert a strong influence over phenotype and a full appreciation of the factors controlling chondrocyte phenotype stability is central to understanding (a) the mechanisms underlying the cartilage failure in osteoarthritis (OA), (b) the rationale for hyaline cartilage repair, and (c) the strategies for improving the engineering of resilient cartilage. The focus of this review is on the factors involved in, and the importance of regulating, chondrocyte morphology and volume as key controllers of chondrocyte phenotype.

**Recent Findings:**

The visualisation of fluorescently-labelled in situ chondrocytes within non-degenerate and mildly degenerate cartilage, by confocal scanning laser microscopy (CLSM) and imaging software, has identified the marked heterogeneity of chondrocyte volume and morphology. The presence of chondrocytes with cytoplasmic processes, increased volume, and clustering suggests important early changes to their phenotype. Results from experiments more closely aligned to the normal physico-chemical environment of in situ chondrocytes are emphasising the importance of understanding the factors controlling chondrocyte morphology and volume that ultimately affect phenotype.

**Summary:**

An appreciation of the importance of chondrocyte volume and morphology for controlling the chondrocyte phenotype is advancing at a rapid pace and holds particular promise for developing strategies for protecting the chondrocytes against deleterious changes and thereby maintaining healthy and resilient cartilage.

## Cell Volume and Morphology—Critical Regulators of Chondrocyte Phenotype

Articular chondrocytes in healthy adult articular cartilage are typically quiescent, highly differentiated cells which, through a balance between anabolism and catabolism of matrix constituents, maintain a resilient extracellular matrix (ECM). For this, phenotypic stability is essential as the chondrogenic type normally synthesises a tough, basket-weave matrix principally comprised of collagen type II and aggrecan. However, chondrocytes are phenotypically unstable and, with relatively little prompting, will undergo de-differentiation resulting in the production of very different extracellular proteins that incorporate into the matrix leading to inferior mechanical properties. For example, the transition from a chondrocytic to a fibroblastic phenotype may occur with a dramatic change in cell shape, cytoskeletal structure, and cell metabolism leading to the increased synthesis of collagen type I and small proteoglycans (e.g. decorin), which are not restrained within the collagen matrix, leading to a weak and mechanically incompetent ‘repair’ fibro-cartilaginous tissue [1]. Alternatively, chondrocytes may take a differentiation route similar to that of chondrocytes in the growth plate, producing collagen type X, markers of hypertrophic chondrocytes, and alkaline phosphatase (ALP), which collectively do not maintain cartilage integrity [2]. The phenotypic instability of chondrocytes that occurs with changes to cell volume and morphology is a major feature and problem in the development of osteoarthritis (OA) [3] and also in abnormal cartilage repair. The maintenance of a chondrocytic phenotype to produce a resilient hyaline-like matrix is also crucial in tissue engineering strategies [4••].

Recent studies have identified early changes to human chondrocyte morphology and volume occurring *before* marked cartilage degeneration and loss, and thus might be important for understanding early stages of tissue failure. In other cell types, the effective control of cell volume and morphology are recognised as fundamental regulators of cell behaviour and metabolism [5, 6]. Changes to chondrocyte volume and shape could be linked either directly or indirectly to the phenotypic plasticity of chondrocytes. Thus, a better understanding of these chondrocyte properties could identify targets for slowing down the pathological changes so as to maintain the chondrocytic phenotype and protect the production of the hyaline matrix with required load-bearing function.

## The Importance of Chondrocyte Shape in Controlling Matrix Metabolism

There is a close relationship among articular chondrocyte properties determining the shape, the cytoskeleton, the chondrogenic phenotype, and the metabolism of hyaline-like cartilage ECM molecules. For example, during the culture of freshly-isolated articular chondrocytes on 2D monolayers, cell morphology changes dramatically from the ‘smooth’ elliptical and spheroidal shapes of chondrocytes present in healthy cartilage [7, 8] to a spreading fibroblastic morphology [9]. This can be restored towards the chondrogenic morphology and phenotype by stimulating a return in cell shape, for example, by culturing in agarose, alginate, or high-density pellets, or on a hydrogel surface [see [10–12]]. Importantly, the re-differentiation of cells with a fibroblastic morphology towards the chondrocytic phenotype can be promoted with drugs which destabilise the cytoskeleton (e.g. dihydrocytochalasin [13, 14]). A crucial role for the cytoskeleton has been identified in primary chondrocytes in situ, in which the arrangement of actin in a cortical ring is necessary for the stability of the chondrogenic phenotype and also for the re-expression of the chondrogenic phenotype following de-differentiation [15]. In 2D culture, parallel bundles of actin (F-type) stress fibres form, whereas de-polymerisation of these fibres promotes chondrogenesis. The architecture of the actin cytoskeleton, its polymerisation status (globular (G):fibrous (F)), and its links to focal adhesion complexes control key signalling molecules that determine whether the chondrocyte phenotype will be instructed to pursue a fibroblastic or a chondrogenic route [16].

During the morphological transformation associated with this de-differentiation process, there are many changes to cell behaviour including decreased expression of the chondrogenic transcription factor SOX9 (SRY-type high-mobility group box-9) and suppressed production of cartilage-specific matrix molecules type II collagen (encoded by *COL2A1*) and aggrecan (encoded by *ACAN*). However, the gene expression and synthesis of fibro-cartilaginous constituents (e.g. type I collagen (*COL1A1* and *COL1A2*)) are markedly increased [17] showing a shift to a fibro-cartilaginous (fibroblastic) phenotype. This is associated with the upregulation of cytokine (e.g. *IL-1β*) genes [18] and even a short exposure of cultured chondrocytes to IL-1 results in marked changes in chondrocyte morphology, phenotype, and matrix metabolism [19]. Treatment with cytokines results in reduced levels of an endogenous GTPase (GTP-Cdc42), leading to decreased *COL2A1* and *ACAN* expression, and increased matrix metalloproteinase-13 (MMP-13). The development of a fibroblastic morphology, associated with actin stress fibres due to cytokine treatment, could be reversed with cytochalasin D, emphasising the plasticity of this process [19]. Significantly, the modification of the actin cytoskeleton alone does not appear to control the full chondrogenic phenotype [20]. Thus, changes to the organisation of other cytoskeletal components may have different effects on matrix metabolism; for example, tubulin polymerisation reduces the production of IL-1β and protease gene expression in primary chondrocytes [21], and disruption of the vimentin network associated with OA has been described [22]. Thus, while alterations to chondrocyte shape strongly suggest a change in phenotype, it is probably not cell shape per se that controls chondrogenesis but, more likely, the complex organisational state of the chondrocyte cytoskeleton and its interaction with second messenger pathways [16].

## A New Look at Cartilage—Imaging In Situ Fluorescently-Labelled Chondrocytes by Confocal Scanning Laser Microscopy

**Fig. 1:**
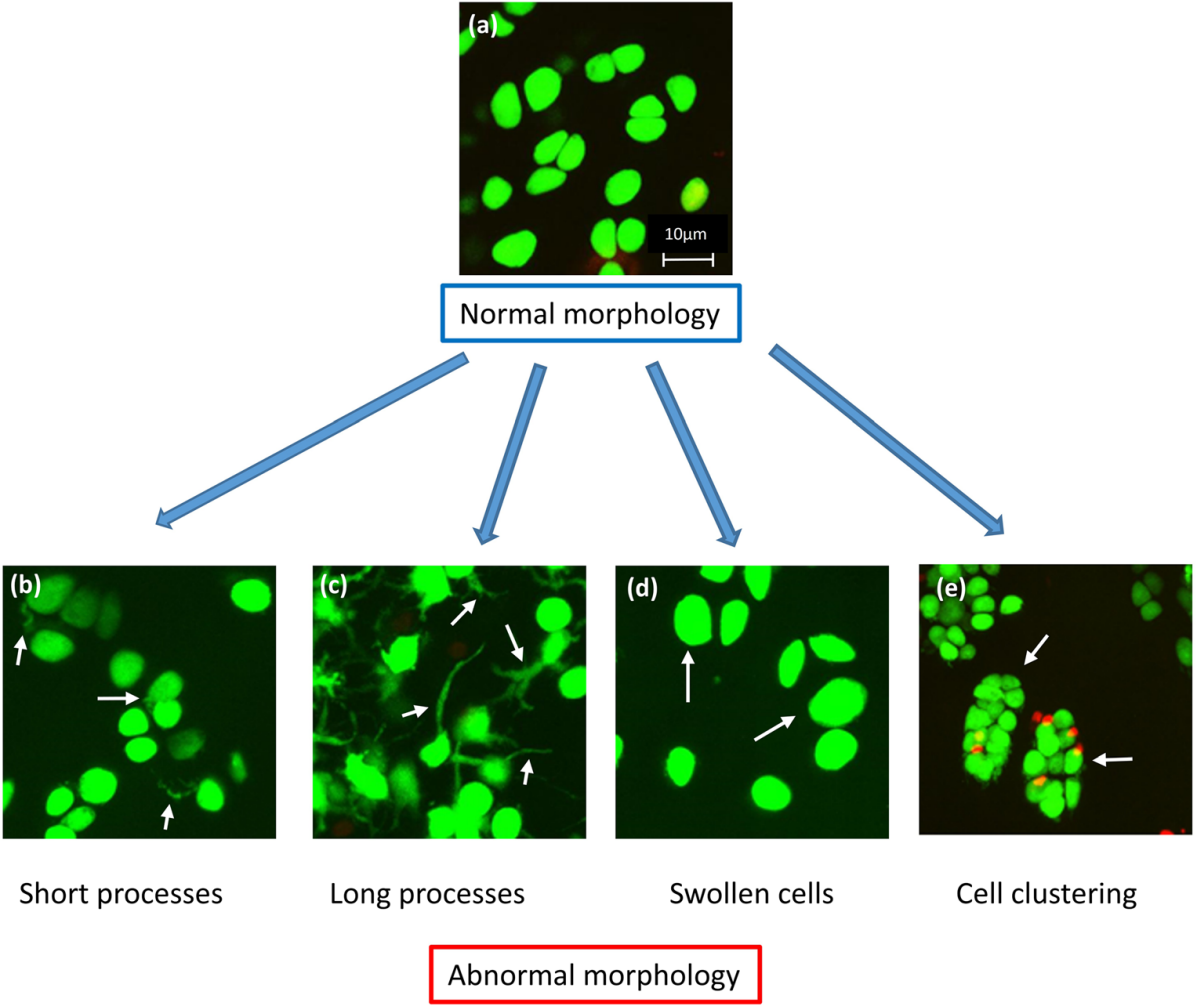
Examples of the heterogeneity of in situ chondrocytes in human femoral head cartilage. The five principal morphologies of human chondrocytes are illustrated: (a) cells with normal elliptical/rounded morphology (grade-0 cartilage), (b) cells with short cytoplasmic processes (up to 10 μm; grade-0 cartilage), (c) cells with long cytoplasmic processes (over 10 μm; grade-1 cartilage), (d) swollen chondrocytes (volume over approx. 1000 μm^3^; grade-0 cartilage), and (e) chondrocyte clustering (grade-1 cartilage). The chondrocytes shown were principally in the superficial zone (SZ). Full-depth osteochondral explants from human femoral heads obtained from femoral neck fracture with ethical permission and patient consent, were incubated with *CMFDA* (5-chloromethyl-fluorescein diacetate) and propidium iodide (10 μM each), prepared for confocal scanning laser microscopy (CLSM) and imaged in the axial direction (i.e. viewed down onto the cartilage surface using a × 40 0.8 NA water-immersion lens) as described [27•]. Chondrocytes are labelled green or red for living or dead cells, respectively. Representative images of chondrocyte morphology are taken from images in Karim et al. [27•] The scale bar shown in (a) is the same for all panels

Early conventional histological studies identified the general features of hyaline articular cartilage and highlighted the topographical arrangement and marked heterogeneity of chondrocyte morphology with depth [7]. However, with the advent of advanced microscopic imaging, which did not require the fixation or the dehydration necessary for histology with associated shrinkage artefacts [23], detailed cellular features have been revealed, which may be important for a fuller understanding of chondrocyte behaviour in normal and degenerate cartilage. Specific fluorescent labelling of the cytoplasmic space and components of living in situ chondrocytes within their unperturbed native ECM has been used with confocal scanning laser microscopy (CLSM [24]) and 2-photon laser scanning microscopy (TPLSM [25]) to produce high-resolution 3D images. Combined with imaging software, these techniques have allowed the study of chondrocyte morphology and volume within relatively thick unperturbed osteochondral explants of non-degenerate and increasingly degenerate (OA) grades of human cartilage [8, 26, 27•]. In addition to the ‘classical’ morphology and chondrocyte clustering observed with standard histology, these studies have identified 4 other chondrocyte morphologies identified by changes to chondrocyte volume and appearance in the form of cytoplasmic processes (Fig. 1). It seems likely that these cells are undergoing changes in phenotype which are potentially associated with damaging changes to matrix metabolism.

CLSM images of in situ chondrocytes [8, 27•] (Fig. 1) have demonstrated that human chondrocyte morphology is more varied than normally described in the cartilage literature. Approximately half of the chondrocytes within macroscopically normal, aged human tibial plateau cartilage have fine cytoplasmic processes usually extending beyond the pericellular matrix (PCM) or lacunar space into the inter-territorial matrix [8]. Studies on human femoral head cartilage also identified abnormal chondrocyte morphology and classified the cytoplasmic processes based on their length and number per chondrocyte [27•]. In grade-0 (non-degenerate) cartilage, the majority of chondrocytes were single and morphologically normal, and topographically arranged as described [7]. However, clusters (containing 3 or more chondrocytes within the lacunar space) were occasionally observed in the superficial zone, and 15–25% of the cell population exhibited at least one cytoplasmic process of approximately 5 μm in length [27•]. The percentage of chondrocytes with these processes, the number of processes per cell, and the average length of the processes all increased significantly in the superficial zone (SZ) in grade-1 compared with grade-0 cartilage. Notably, chondrocytes with cytoplasmic processes were observed in axial (i.e. ‘top down’) views [27•] ruling out the possibility that the processes were an artefact from tissue injury or sampling [8]. Similar abnormal ‘fibroblastic-like’ chondrocytes have been observed under light or electron microscopy of fixed samples of normal and fibrillated human knee cartilage [28, 29] and human femoral head cartilage, albeit overlaid with pannus [30]. For grade-0 tissue taken from tibial plateau cartilage, there did not appear to be a correlation between the percentage of abnormal chondrocytes and patient age [31]. These processes are not related to the chondrocyte primary cilium, which is considerably shorter (1–2 μm) and requires specific cytoskeletal fluorescent probes for visualisation [32].

It seems likely that the development of abnormal chondrocytes is associated with alterations to the PCM, which contains abundant proteoglycans and is the exclusive location of collagen type VI [33]. The PCM also determines the cell’s mechanical environment and profoundly influences cell behaviour and metabolism through the transduction of biochemical and biomechanical signals [34•]. Changes to the content and structure of the type VI collagen have been observed around chondrocytes in OA [35], particularly those with cytoplasmic processes [31].

It is possible that direct mechanical damage to the PCM leads to weaknesses or fissures through which cytoplasmic process(es) can protrude. However, injury to the PCM could be initiated by chondrocytes themselves as there is a close relationship between the development of abnormal chondrocyte morphology and increasing levels of cell-associated IL-1β as assessed by fluorescence immunohistochemistry and quantitative CLSM imaging on the same cells [31]. High levels of this cytokine were associated with chondrocytes possessing large numbers of short cytoplasmic processes. The IL-1β may be a product of chondrocytes [31] and/or be part of a more general low-grade inflammatory response in the surrounding joint tissues [36, 37]. This cytokine stimulates the release of degradative enzymes (e.g. MMP-13) [38] which could weaken the PCM locally, thereby producing areas of mechanical fragility and providing an avenue(s) for a process(es) to extend, potentially accelerating the development of the fibroblastic phenotype. From numerous studies on the relationship between chondrocyte shape and matrix metabolism, there are likely to be a wide range of other changes still to be investigated. In any event, the presence of these delicate cytoplasmic processes could pre-dispose abnormally-shaped chondrocytes to injury as a result of mechanical loading. This could account, in part, for the chondrocyte hypo-cellularity which may be a cause or effect of OA [3].

Chondrocytes with these abnormal cytoplasmic processes have not been observed in non-degenerate animal cartilages (rat, bovine, equine [8]). However, if cartilage is mechanically injured as a result of scalpel cutting or impact loading and cultured in the presence of fetal calf serum (FCS) or synovial fluid, chondrocytes developed cytoplasmic processes similar to those observed in human cartilage [39, 40]. Interestingly, despite the presence of FCS, raising the osmotic pressure of the culture medium prevents the development of these cytoplasmic processes [39]. It is possible that hyperosmolarity stimulates the production of the SOX9 genes, which maintain the chondrocyte phenotype [41] (in spite of the growth/morphogenic factors in FCS). CLSM studies on fluorescently-labelled chondrocytes show similar processes also occasionally present after 7 days in stiff (2%) 3D agarose cultures containing FCS. However, their incidence rises markedly in soft (0.2%) agarose gels [42]. It is possible that the penetration of the growth/mitogenic factors in FCS into cartilage and stiff gels is severely restricted, but when the matrix or gel is weakened (by scalpel injury or culture in soft agarose gel) these factors can penetrate and stimulate chondrocyte morphological changes. An interesting parallel is that the extensive clustering of chondrocytes, which characterises the later stages of OA, arises from the increased access of the growth factors due to cartilage fibrillation [43].

## Cartilage Osmolarity, Swelling in Early OA, and Chondrocyte Volume

The osmotic environment of connective tissue cells, including articular chondrocytes, is complex, varies under physiological and pathophysiological conditions, and is therefore probably unique to animal cells. It is controlled by the proteoglycans (PGs), principally aggrecan, in the ECM which, because of their strong negative charge, influence cation and anion distributions and thus the interstitial osmolarity and ionic composition surrounding chondrocytes. The osmolarity varies from approximately 350 mOsm in the SZ of human articular cartilage to around 450 mOsm in the mid-zone (MZ) [44]. The osmolarity of the synovial fluid bathing the cartilage is also considerably higher than that of most body fluids (e.g. serum) and standard tissue culture media, which are both around 280–320 mOsm [45]. In normal joints, synovial fluid osmolarity is estimated to be around 404 mOsm, which decreases with exercise to 301 mOsm and is 297 mOsm in osteoarthritic joints and ~ 280 mOsm in rheumatoid arthritis [46, 47]. A similar situation occurs in the intervertebral disc, as in healthy tissue the extracellular osmolarity varies from 430 (iso-osmotic) to 500 mOsm (hyper-osmotic) [48], but is less with disc degeneration (~ 300 mOsm) [49]. The regulation of cell volume of isolated or in situ cells under these anisotonic conditions is very important for maintaining optimal matrix metabolism [45, 48, 50]. For example, the isolation of chondrocytes from bovine cartilage, where the osmolarity ranges from 380 to 480 mOsm, and transfer to standard tissue culture medium (typically 280–320 mOsm) will cause rapid chondrocyte swelling and marked decreases in GAG and protein synthesis rates [45].

Chondrocytes in situ in normal and degenerate cartilage are freely permeable to water [51–53]. The aquaporin (AQP) water channels AQP1 and AQP3 [54, 55] are very important for mediating the responses to changes in extracellular osmotic pressure, with alterations to chondrocyte volume occurring within minutes following suspension of osteochondral explants in anisotonic media [53]. At rest, the volume ‘set point’ of chondrocytes is determined by the prevailing tissue osmolarity and is associated with optimal matrix biosynthesis [45]. Chondrocyte volume may change in the short term as a result of diurnal static loading patterns leading to fluid expression and raised osmolarity; however, these changes are relatively small (~ 5%) and focal [56]. In contrast, more marked changes may occur, for example, as a result of increased cartilage hydration in OA or tissue water loss with ageing [57]. The striking increase in cartilage hydration in OA has been described as one of the earliest macroscopic changes occurring with cartilage degeneration. In the human femoral head, the water content, expressed as the ratio of cartilage water weight:dry weight, increases by ~ 60% [57]. Cartilage hyper-hydration is also observed in the canine DMM (destabilisation of the medial meniscus) model of OA [58] and in a spontaneous model of OA [59]. Cartilage swelling occurs before cartilage loss and probably arises from damage to the collagen network (possibly collagen type IX) leading to a reduction in its elastic restraint allowing the glycosaminoglycans to increase their hydration [60]. This will reduce tissue interstitial osmolarity [45] and increase chondrocyte volume [8]. It also seems probable that the reduced cartilage resilience will cause greater variations in tissue osmolarity and thus chondrocyte volume with otherwise normal loading cycles.

Measurements of the volume of fluorescently-labelled in situ chondrocytes within relatively unperturbed osteochondral explants from non-degenerate and degenerate human cartilage have been possible using quantitative CLSM imaging [8]. These explants are required for study, since removal of the cartilage from the attached bone may cause further swelling of cartilage and chondrocytes because of damage to the collagen network characteristic of degenerate cartilage [61]. In tibial plateau cartilage, chondrocyte volume progressively increases in all zones with degeneration, but because of the very wide range of cell volumes, not all of the differences are statistically significant [8]. The increase in the mid-zone (MZ) is most significant, rising on average from 522 μm^3^ in grade-0 cartilage to 990 μm^3^ in grade-3 cartilage with wide ranges in cell volumes for all cartilage grades. For example, in the MZ of grade-0 cartilage, there some small cells (200–300 μm^3^) and some very large cells (> 1000 μm^3^), the range of volumes probably reflecting local aggrecan concentrations.

This increase in cell volume could be related to changes to a hypertrophic phenotype, associated with increased expression of hypertrophy markers (type X collagen, MMP-13) in human OA cartilage and in animal models of joint instability [62, 63]. However, this should be viewed in the context of the volume increases occurring in hypertrophic chondrocytes in the growth plate. For example, in the rat growth plate, chondrocyte volume increases from 1000 μm^3^ in the proliferative zone to approximately 12,000 μm^3^ in the hypertrophic zone [23]. Although the volume change in human OA cartilage is not as dramatic, it is possible that a relatively small and focal but long-term uncompensated increase would be sufficient to stimulate the progressive development of a hypertrophic phenotype with associated changes in matrix metabolism. In any event, chondrocyte swelling arising from the reduced tissue osmolarity in OA is likely to markedly increase the risk of cell damage or death as a result of mechanical loading, since chondrocytes in swollen cartilage are highly sensitive to a single impact load. In contrast, raising medium osmolarity, which results in cell shrinkage, is chondroprotective [64].

Another situation, in which changes to cell volume might occur in normal and OA cartilage, may result from cell proliferation or ‘clustering’. This is a property of some normal (non-degenerate) cartilages, for example, in the SZ of human ankle cartilage, where horizontal cell clustering (‘strings’) occurs parallel to the surface [65, 66]. However, large clusters of spheroidal chondrocytes (20 or more cells per cluster) located within substantial lacunar spaces are well-established histological features of OA [43]. These are normally observed near-surface fissures or fibrillations [43] partly as a result of the penetration of cytokines/growth factors from the synovial fluid into the weakened and damaged matrix stimulating cell proliferation. In mildly degenerate human femoral head cartilage studied with quantitative CLSM, the volume of clusters and the number of cells per cluster suggest that the increase in the size of clusters is primarily due to chondrocyte proliferation rather than cell swelling/hypertrophy. The relatively small (~ 20%) increase in chondrocyte size suggests some degree of volume regulation or mechanical restraint by the PCM, whereas the overall volume of clusters increases by more than 3-fold [27•]. While the swelling of chondrocytes within clusters is modest, they are nevertheless associated with increased levels of collagen type X, MMP-13, and other matrix degradative enzymes which are characteristics of the hypertrophy of chondrocytes in OA [43, 62].

Changes to the extracellular ionic composition and osmolarity have profound effects on ECM metabolism by connective tissue cells. This has been demonstrated in articular cartilage and intervertebral disc explants, isolated chondrocytes, and chondrocytes cultured in alginate [45, 67–69], as well as isolated intervertebral disc cells [70]. In articular cartilage, variations in medium osmolarity above or below that in native non-degenerate cartilage, inhibit optimal matrix GAG and protein synthesis [45]. Alterations to osmolarity also closely regulate the expression of genes encoding hyaline cartilage ECM proteins, including collagen type II (*COL2A1*) and aggrecan (*ACAN*) [41] through the cartilage master regulator SOX9 [71, 72]. Raised osmolarity increases SOX9 mRNA stability and SOX9 protein production [41] and SOX9 also suppresses ADAMTS (A Disintegrin And Metalloproteinase with Thrombospondin Motifs) activity [73]. This chondrogenic transcription factor is therefore crucial for the maintenance of normal cartilage viability. Fukui et al. [74], using laser micro-dissection, demonstrated that, within OA cartilage, the expression of cartilage matrix genes was significantly correlated with SOX9 expression. While there have been many studies investigating the control of chondrocyte matrix metabolism, particular caution should be taken when interpreting data utilising experimental protocols where chondrocytes are isolated and cultured under conditions different from those experienced within their normal ECM. Chondrocyte isolation using enzymes and subsequent culture in standard media potentially exposes cells to changes in their physico-chemical environment. Thus, there could be a rapid reduction in osmolarity, changes to ionic constituents (e.g. Na^+^ K^+^, Ca^2+^, H^+^, HCO_3_^−^), exposure to unfamiliar factors (e.g. serum), a raised pO_2_, and a very different mechanical environment [45, 75]. It is therefore hardly surprising that under some culture conditions, the metabolism of components of the complex ECM may be compromised. Whether such changes persist as a new matrix forms around the cells with longer term culture, in most cases, has not been addressed. However epigenetic changes in long-term monolayer cultures of human chondrocytes obtained from non-OA human cartilage have been described [76]. The increased DNA methylation observed dampened the transcriptional activities of the *MMP13* and *IL-1β* genes. This raises the interesting possibility that epigenetic inhibitors could have therapeutic potential by suppressing the activity of these two catabolic genes which play a key role in OA progression [36–38].

## Volume Regulation by Chondrocytes

In view of the close relationship between external osmolarity, chondrocyte volume, and matrix metabolism, it is important to clarify the role of the volume-sensitive membrane transport pathways and channels for osmolytes, as they are key components of the signal transduction pathway. A range of studies have demonstrated that there are marked differences in the responses of chondrocytes depending on the rate of change in osmolarity. The majority of research has been performed by challenging chondrocytes with a rapid (*acute*—seconds or minutes) hypo- or hyper-osmotic challenge. This is relatively straightforward experimentally, and with pharmacological agents and other techniques, the important membrane transporters and ion channels involved in restoring cell volume towards that present initially can be identified. However, such rapid changes in osmolarity and cell volume are unlikely to occur physiologically or pathophysiologically, as changes in tissue osmolarity, resulting from cartilage loading or from the tissue swelling in OA, will have a considerably longer time course, resulting in a *chronic* osmotic challenge and lasting from days to months or even years.

### Acute Osmotic Challenge

In situ and isolated chondrocytes are very responsive to changes in interstitial and medium osmolarity. Over a range of extracellular osmolarity, this ‘passive’ response (i.e. under conditions to minimise the ‘active’ contributions of the membrane transporters and channels that control cell volume) of in situ and isolated chondrocytes follows that expected of a perfect osmometer [51]. In other words, when medium osmolarity is varied from 280 to 600 mOsm, the PCM, ECM, and intracellular cytoskeletal elements do not limit the change in volume, and the cells behave freely as if no osmotic restriction elements are present. However, further reducing osmolarity to below about 200 mOsm, corresponding to hyper-hydrated cartilage, results in little further swelling of chondrocytes in normal cartilage, whereas cells in OA cartilage continue to swell. This suggests that, in normal cartilage, the resilient matrix prevents additional cell swelling, whereas in degenerated cartilage, the surrounding PCM is mechanically weakened probably as a result of structural changes to collagen type VI microfibrils [31, 33, 77, 78], thereby permitting further swelling. The rapid change in the volume of articular chondrocytes in response to variations in osmolarity arises from the high permeability of the ECM to water, and the presence of AQP water channels on chondrocytes. Of interest is a report that AQP1 gene expression levels are increased by around 39-fold in chondrocytes of human knee OA cartilage compared with cells in ‘microscopically’ intact cartilage [78]. This could be a cellular response associated with the increased cartilage hydration in OA [60].

The rapid increase in cell volume resulting from the reduced extracellular osmolarity may be followed by ‘active’ (i.e. involves membrane transporters/channels) regulatory volume decrease (RVD), which restores cell volume towards normal [52]. Approximately 50% of isolated chondrocytes demonstrate the RVD response to acute osmotic challenge [79, 80], in contrast to chondrocytes in situ, where > 90% of the cells in all zones of bovine articular cartilage demonstrate strong RVD [52]. This may be because the chondrocyte isolation procedure using enzymes and unphysiological and anisotonic culture media, has damaged the cells. On the other hand, raising medium osmolarity causes rapid chondrocyte shrinkage; however, the recovery of cell volume (by regulatory volume increase, RVI) by bovine chondrocytes in situ is relatively slow and only partially complete over a comparable time period [81]. The lack of a need for a strong RVI response is perhaps not surprising, given that cartilage water loss and thus the raised interstitial osmolarity would be restored to normal anyway as part of the diurnal loading cycle on the joints. The membrane transporters involved in RVI have not been studied in as much detail as those for RVD; however, there is evidence that the Na^+^/K^+^/2Cl^−^ (NKCC) co-transport pathway which is sensitive to the ‘loop’ diuretic bumetanide is primarily involved [81]. It is interesting that, in the growth plate, the activation of the bumetanide-sensitive membrane transporter NKCC1 is considered a major driver of chondrocyte swelling and the hypertrophic phenotype [82]. Clearly, for a cell to swell, not only do the transporters and ion channels mediating the accumulation of osmolytes in the RVI response have to be stimulated, but those which are involved in RVD must be inhibited; otherwise, no net volume change may occur.

### Chronic Osmotic Challenge

The responses of chondrocyte volume, the membrane transporters and ion channels, and cell metabolism to *gradual* changes in extracellular osmolarity are very different compared with those involved in acute osmotic change. This is mainly because the corresponding osmolyte movements mediated by the volume-sensitive membrane transporters and ion channels keep pace with the volume changes through continuous volume adjustment resulting in limited changes to cell volume. This is termed iso-volumetric regulation (IVR), and although it has been studied in detail in other cell types (e.g. [83]) and despite its potential importance, it has not yet received sufficient attention in chondrocyte volume regulation. An interesting study on freshly isolated bovine articular chondrocytes reduced osmolarity from 350 to 140 mOsm either rapidly (5 min) or gradually (over 180 min). The acute hypo-osmotic challenge caused rapid cell swelling followed by robust RVD, whereas the gradual change caused a much smaller increase in cell volume, with only a weak RVD response [80]. Importantly, up- or downregulation of transporter expression may occur as well as changes to cell metabolism leading to enhanced synthesis or degradation of intracellular osmolytes (e.g. sugars, polyols, amino acids) to compensate for the changes in volume. For example, transcription of the non-essential amino acid taurine (2-aminoethanesulphonic acid) transporter gene in chondrocytic cells is upregulated by hypertonic conditions [84] leading to osmolyte accumulation. In response to changes in osmolarity, these osmo-protective molecules may moderate changes to cell volume under anisotonic conditions and potentially confer cyto-protective and anti-inflammatory effects [85]. In human intervertebral disc cells following hyper-osmotic challenge, the gene expression profile identified 42 genes that were significantly changed, including those involved in cytoskeletal remodelling and ion and osmolyte transport [86].

## Activation of Volume-Sensitive Osmolyte Pathways in Anisotonic Conditions

The direct activation of membrane transporters and ion channels that mediate osmolyte movement and volume changes can occur *without* a change in intra- or extracellular osmolarity. A good example is in the growth plate where this drives chondrocyte swelling, hypertrophy and ultimately cell death, and the zone of calcification where osteoblasts form new bone. The stimulation of the NKCC cotransporter drives cell swelling [82] and it is necessary that RVD transporters and channels are suppressed to allow the volume increase to proceed. Chondrocyte hypertrophy in the growth plate is a form of ‘programmed cell death’ in the sense that the cells must be signalled to die through swelling and lysis, in order to leave behind the structural elements which form the advancing bone front. However, this process in the growth plate is often described, perhaps inaccurately, as ‘apoptosis’ which classically involves a *decrease* in cell volume, clearly identified as a key step in the process [87, 88]. Notwithstanding the apparent confusion in the literature, the shrinkage occurring in ‘classical apoptosis’ occurs through the direct activation of K^+^ and Cl^−^ channels leading to the loss of ions with associated water and cell shrinkage (‘Apoptotic Volume Decrease’, AVD, or ‘normotonic’ cell shrinkage) [89]. In cartilage, the stimulation of chondrocyte volume-sensitive Cl^−^ channels contributes to cell shrinkage and may accelerate cell death through apoptotic-like pathways [90]. As the Cl^−^ gradient is normally into the cells, the activation of these channels probably plays a ‘permissive’ electrochemical role in balancing the charge of the main osmolyte (K^+^), and allowing it to leave the cells down its gradient, thereby causing cell shrinkage. While AVD is identified as an essential step in classical apoptosis required for cell shrinking, the membrane transport pathways that may otherwise protect against cell shrinking (e.g. NKCC1) are inactivated [91]. Thus, it is the *balance* between the two opposing volume-regulatory responses (RVD and RVI) that may ultimately determine the change to cell volume and, if required, its maintenance at a new ‘set point’. While there has been considerable focus on cell volume [see [50], there is a realisation that it might not be volume per se that is the key regulator of cell metabolism, but cellular composition. For example, a reduction in intracellular K^+^ concentration ([K^+^]_*i*_) in the absence of a volume change has been proposed as an essential step in the apoptotic pathway [83, 92].

Interest in chondrocyte ion channels is developing rapidly because they are involved in fundamental aspects of cartilage biology and implicated in disease processes potentially offering specific targets for novel and pre-existing pharmacological and biological agents. Knowledge of the wide range of chondrocyte ion channels has been obtained from electrophysiological and microarray data with important developments in our understanding of the wide range of roles of K^+^ and Cl^−^ channels, including cell volume regulation, cell proliferation, differentiation, migration, and cell death pathways [50, 93, 94] and TRP channels (see [95, 96]). For example, a recent report suggests that, in the rabbit anterior cruciate ligament transection model (ACLT), activation of a chondrocyte Cl^−^ channel occurs prior to gross cartilage damage [97]. It is possible that the joint instability has caused activation of the channel either directly through mechanotransduction or indirectly through cartilage swelling, thereby raising the risk of chondrocyte death via apoptotic pathways and hypo-cellularity leading to cartilage degeneration [98, 99]. However, the chondrocyte death does not appear to be the result of ‘classical’ apoptosis which occurs following cell shrinking; instead, it is characterised by increased amounts of Golgi, ER, primary lysosomes, and autophagic vacuoles and considerable blebbing/extrusion of cytoplasmic components—the process of ‘chondroptosis’ [100, 101]. Furthermore, chondrocyte swelling, and not shrinkage, appears to correlate with human cartilage degeneration in OA [8]. While this chondrocyte death pathway is a possible mechanism for the cartilage degeneration in this injury model, it is unknown whether this is the main mechanism in human OA. There is a continuing debate about the suitability of animal models of joint instability which have been considered to more accurately reflect post-traumatic osteoarthritis (PTOA) [102–104] than ‘primary’ idiopathic human OA. While these models could give insights into PTOA, this disorder only contributes about 12% of all cases of human OA [43]. In animal models, the initial insult is clearly defined and the degeneration of the thin cartilage of relatively young animals is very rapid compared with human OA. While the end-stage pathology involving cartilage fibrillation and loss is often considered to be broadly similar to idiopathic primary OA, the initiating and earlier sequences of events could be different.

The cation-selective chondrocyte transient receptor potential (TRP) channels have important roles as controllers of chondrocyte matrix metabolism, cell volume, and inflammatory/pain responses, through their regulation of the intracellular Ca^2+^ concentration [96]. Many TRP channel types are sensitive to chondrocyte volume and morphology and the finding that there are changes to expression levels of TRP channels in native and cultured chondrocytes from OA cartilage [105] has stimulated interest in their potential role in the progression of cartilage degeneration in animal models. Recent work has focussed on the vanilloid (TRPV) sub-family as the TRPV4 channel could be particularly important in the transduction of the mechanical/osmotic loading of articular cartilage by permitting the generation of intracellular Ca^2+^ transients. In a mouse model, deletion of TRPV4 channels leads to cartilage degeneration; [106] however, loss of this channel does not prevent cartilage failure in the DMM model but interestingly reduces the severity of age-related cartilage degeneration in a mouse model of OA [107]. Evidence is accumulating that TRPV4 could be an important regulator of chondrogenic differentiation as it shows gene expression patterns similar to those of *COL2A1* and *ACAN*, and the increased Ca^2+^ influx mediated by phorbol esters upregulates the SOX9 transcription factor [108]. *Trpv4*^*−/−*^ mice spontaneously develop cartilage degeneration, and it has been suggested that the absence of this channel removes the chondroprotective mechano-osmotic sensing capacity of chondrocytes, which is fundamental for normal cartilage biology [106]. TRPV6 may also act as a chondroprotective factor, as knockout mice exhibit cartilage degenerative changes, including GAG loss, fibrillation, and eburnation [109]. This chondroprotective role of some of the TRPV channel types suggests that they could defend the chondrocytic phenotype in the face of mechanical and osmotic challenges, possibly by stimulating SOX9-dependent gene expression levels [110].

## Can We Mimic the Physico-chemical Environment of the ECM and Thus Normal Chondrocyte Volume, Shape, and Phenotype in Culture?

In some respects, research has been rather slow to appreciate that standard tissue culture media and other artificial conditions not typically experienced by chondrocytes are not optimal for normal chondrocyte metabolism and the production of a hyaline cartilage-specific matrix. There are many changes to the physico-chemical environment of chondrocytes when they are released enzymatically from their native extracellular matrix and exposed to inappropriate ‘traditional’ culture medium with an ionic and osmotic composition that is totally different from the extracellular environment of chondrocytes in situ [45]. For cartilage engineering, research efforts are directed towards improving matrix metabolism to favour a hyaline-like ECM, and this appears to be gathering pace as an appreciation of the importance of suitable culture conditions becomes recognised [4]. Attention has been given to the development of in vitro culture methods and medium supplementation to include physiological regulators of chondrogenesis to stimulate cell proliferation and matrix synthesis, while inhibiting hypertrophy and the catabolic responses to cytokines [111]. Chondrocytes are also cultured in 3D scaffolds, which inhibit the formation of actin stress fibres and cell spreading, which are closely involved in the development of the fibroblastic phenotype [11]. The prevention of chondrocyte de-differentiation and the stimulation of hyaline-like cartilage formation has been shown using medium containing serum and growth factors, or ITS (insulin-transferrin-selenium) as a serum substitute [112]. Furthermore, reduced oxygen tension during culture, which promotes the chondrogenic phenotype and reduces oxidative damage [113], increases the differentiation of MSCs towards the chondrogenic lineage [114]. In addition, appropriate mechanical stress is crucial in order for the cells to receive the valid signals for the production of a resilient, load-bearing ECM [115]. Identifying optimal levels of mechanical stress even for chondrocyte cultures, let alone whole joints, is challenging. However, a recent study applying mild dynamic movement to the bovine metatarsophalangeal joint maintained chondrocyte viability and GAG content over 28 days, whereas these declined over the same time period in the static joint model [116].

It is perhaps not surprising that modifying the components of the culture media, and thus chondrocyte volume to make cell composition closer to that experienced in situ, will improve the properties of engineered cartilage. For example, Ylarinne et al. [117] provided evidence of increased cartilage formation by primary chondrocytes cultured in Transwell inserts in hypertonic (with NaCl) high-glucose Dulbecco’s modified Eagle’s medium (HG-DMEM; 25 mM glucose; 390 mOsm) compared with standard DMEM. Sampat et al. [118] demonstrated that primary chondrocyte-seeded constructs achieved a Young’s modulus and GAG content close to that of native immature bovine cartilage using hypertonic culture (400 mOsm NaCl or KCl) compared with ‘isotonic’ culture medium (330 mOsm). Raising the osmolarity of standard differentiation media by 100 mOsm markedly increased the expression of chondrogenic markers of progenitor cells [119]. Recent work has focused on the optimal type of osmolyte and osmolarity necessary to enhance the chondrogenesis of mesenchymal stem cells (MSCs) [120]. Bertram and Krawetz [121] studied synovial fluid mesenchymal progenitor cells (sfMPCs), a cell type which could be involved in cartilage repair/regeneration, varied osmolarity above and below that of tissue culture medium (300 mOsm). They identified effects on markers of chondrogenesis (e.g. SOX9, ACAN, COL2A1) and proposed that sfMPCs retained their elevated chondrogenic potential if they were differentiated at their native osmolarities. It has also been noted that repair tissue formation is stimulated following in vivo cartilage injury, when scalpel injury is performed in the presence of a hypertonic chondroprotective medium compared with ‘normal’ (0.9%) saline which is typically used in orthopaedic procedures [122]. A mathematical model has been developed for evaluating GAG synthesis within cartilaginous tissues, as well as understanding the role of mechanical loading in tissue growth or degeneration. It has also been suggested that it could be useful for designing a bioreactor system with appropriate extracellular environment and mechanical loading conditions for growing tissue at the maximum synthesis rate of the ECM [123].

## Conclusions

Chondrocyte volume and morphology profoundly influence the stability of the chondrocyte phenotype and it is important to understand their involvement in OA pathology, cartilage repair, and cartilage engineering. Changes to cell volume markedly alter the synthesis of the ECM and a chronic increase in volume is associated with the hypertrophic phenotype. Conversely, prolonged and uncompensated cell shrinkage may be a stimulus for cell death pathways. Changes to chondrocyte shape involving cytoskeletal elements and their associated second messenger pathways can stimulate the transition to a fibroblastic phenotype leading to the production of a mechanically weak matrix. Imaging of chondrocytes in situ within relatively non-degenerate human cartilage has identified changes to their shape and volume which could represent early deleterious transitions to a fibroblastic or hypertrophic phenotype. While it is unclear if these are very early steps initiating cartilage degeneration as occurs in OA, or the result of cartilage or other tissue failure due to other causes, the uncompensated changes in phenotype will ultimately have deleterious effects on the normal turnover of the specific matrix molecules essential for mechanically-resilient hyaline cartilage.

The phenotypic stability of chondrocytes is profoundly controlled by a wide variety of factors, including the substrate used for 2D vs 3D culture, osmolarity, and composition of culture media (pO_2_, FCS, etc.). Any or all of these could potentially alter chondrocyte volume or shape and thus signal chondrocytes to develop the fibroblastic or hypertrophic phenotype with the associated production of a mechanically inferior ECM. There are clearly areas of future research that would prove particularly fruitful. For example, a thorough understanding of the link between the various transporters and channels of chondrocytes, the regulation of the intracellular composition, and the biosynthesis of a viable cartilaginous matrix is important. The relationship between chondrocyte shape and matrix metabolism is well established but the signalling pathways, particularly those involving intracellular Ca^2+^, require further detailed study. These research areas are particularly challenging as analyses will probably have to be performed in situ, as chondrocyte behaviour and phenotypic stability will be strongly influenced by the removal of the cells from their native matrix and their culture in an alien environment. A deeper understanding of the subtle early changes to human chondrocyte volume and morphology occurring before overt cartilage degeneration, as described here, is of importance. This may offer targets for correcting the imbalance between anabolic and catabolic hyaline-like matrix metabolism, potentially stimulating cartilage repair and also improving the resilience of engineered cartilage. For OA treatment, such targets for intervention might be more successful than attempting to stimulate the repair of already weakened, fibrillated cartilage.
